# Early thrombolysis in an elderly patient: case report

**DOI:** 10.1186/1471-2318-11-S1-A8

**Published:** 2011-08-24

**Authors:** M Delle Curti, A Ciao, S Di Costanzo, B Lettieri

**Affiliations:** 1 Department of Anaesthesia, Surgical and Emergency Science, Second University of Naples, Italy

## Background

Pulmonary thromboembolism (TEP) is related to the migration of thrombotic material from the systemic venous circulation to pulmonary vascular tree, with obstruction of the pulmonary arterial circulation.

The mortality rate is 30% in non-identifiable forms, and 2-8% in those treated earlier.

The early use of thrombolytics determines the success of the lysis. Thrombolysis is considered a proven treatment in the management of acute, massive forms of TEP that accompany a hemodynamic instability, but differences arise over time, the doses and which thrombolytic use, to minimize the adverse events in compromised patients. Adverse events are: major bleeding, strokes, severe hypotension, allergies, re-thrombosis and reperfusion syndrome.

Contraindications are: absolute (internal bleeding in progress, recent spontaneous intracranial hemorrhage) and relative (interventions Chir. <10 days, stroke <2m., Gastric bleeding <10 days, Major trauma <15 days, Recent CPR, PTL <100,000, poorly controlled severe hypertension, pregnancy).

## Case report

Vincenzo R. 71 years-old, Kg110, is hospitalized for endoscopic pneumatic lithotripsy for an upper right ureter calculosis.

U.S. the lower limbs: thrombosis of the twin vein medial.

**Figure 1:**
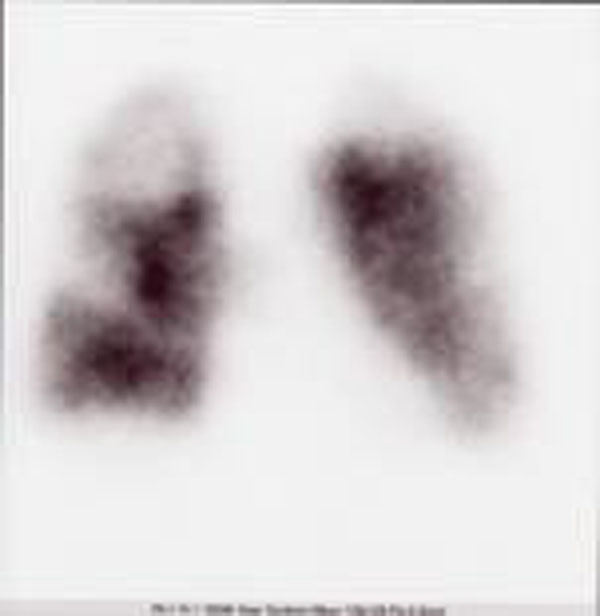
Scintigraphy during the events.

The maneuver, in general anesthesia, extends for 90’. At the awakening, after extubation occurs suddenly, dyspnea, pallor, precordial oppression. The ECG shows: FA with high ventricular response (160 bpm). There is also: PAs to 60mmHg, SpO2 (65%), moist raTes at bases, cyanosis. The blood gas analysis performed on the patient is reported in Fig 1, chest X-rays show a sub-total haziness of both hemithorax by interstitial edema and alveolar perfusion and a scintigraphy performed at 5 hours from the event supports the clinical suspicion of high probability for TEPM in place. D-dimer> 500μg / l confirmed the diagnosis. There are criteria for thrombolysis practice which lack the absolute and relative contraindications. After baseline control of coagulogramma we decide to give rtPA in less time than the traditional protocol (90’ vs 120') with 40 mg iv bolus within 15' with an additional 60 mg infusion in 250ml NaCl 0,9%for 80'. The fall of the PTL and FDP are considered tolerable, bleeding seems to be minimal. On the third day we look at weaning with good spontaneous respiratory activity.

**Figure 2:**
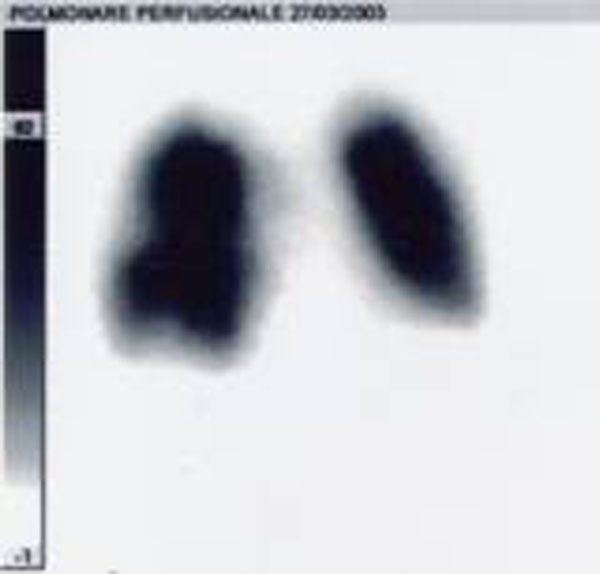
Scintigraphy after the events.

**Figure 3:**
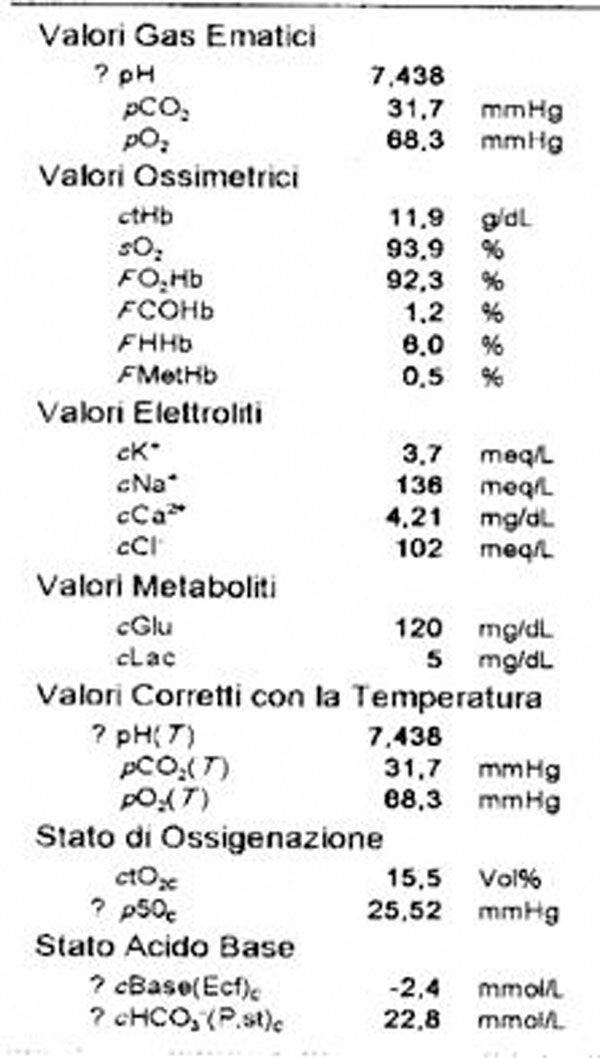
emogasanalysis during the events

**Figure 4:**
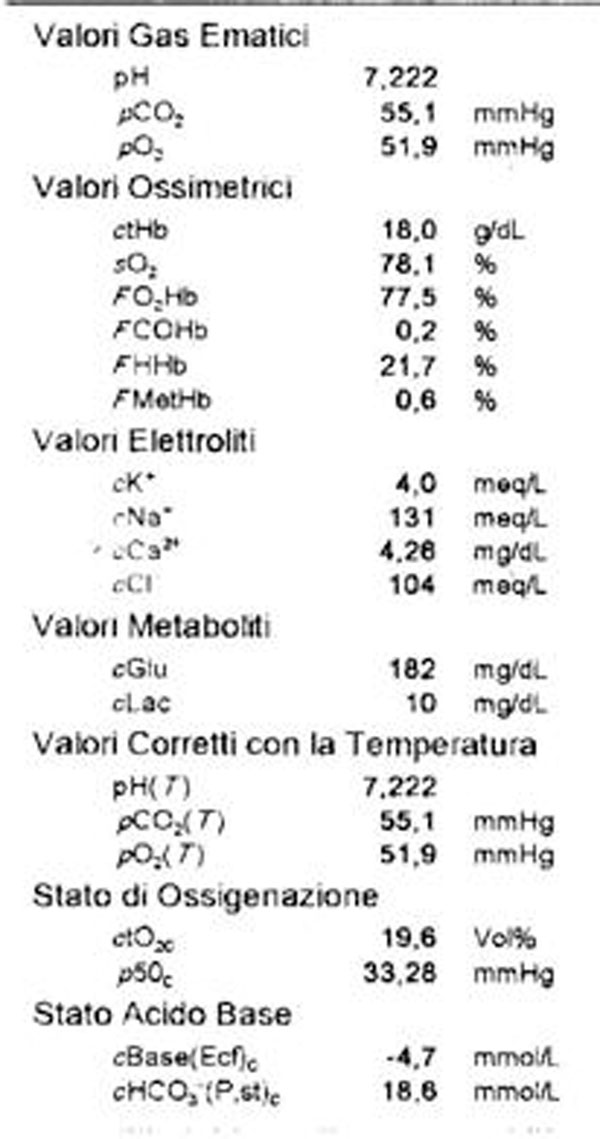
emogasanalysis after three days

## Conclusions

The case report is characterized by the presence of the most frequent risk factors for TEP, DVT, obesity, and by clear indications for thrombolysis: severe hemodynamic instability, the absence of contraindication, early diagnosis and therapy (<8h). This allowed the procedure to obtain a good recovery accelerated thrombolysis and clinical scintigraphy without major bleeding complications.
